# New York State Veterinary Medical Society

**Published:** 1891-09

**Authors:** Nelson P. Hinkley

**Affiliations:** Secretary


*Secretary.*

---

*New York State Veterinary Medical Society.*—The semi annual meeting of the New York State Veterinary Medical Society, was held at the Parlors of the Grand Central Hotel, New York, on Wednesday August 12, 1891. Owing to the delay of members in reaching the city, the hour for calling the meeting to order was extended.

President Morris called the meeting to order at 10 A. M.

A limited number of members responded to the roll call, and several came in later in the day. Letters and telegrams of regret were received from several members of the society and profession. These were also present: W. Horace Hoskins, D. V. S., Secretary of the United States Veterinary Medical Association; Prof. A. Liautard, New York; Roscoe R. Bell, D. V. S., President of the Long Island Veterinary Association; Geo. H. Berns, D. V. S., ex-President of the Long Island Veterinary Association of Brooklyn; and several other prominent members of the profession from New York and Brooklyn.

President Morris delivered an address of a few well-chosen remarks, explaining why the place of meeting had been changed, that it was done to please members of the profession in the eastern part of the State and especially, New York City and Brooklyn, and to endeavor to get their valuable aid and assistance in promoting the matters pertaining to Legislation, and the good of the society and the profession generally, and to give to those desiring to become members of the society an opportunity to do so. Dr. Morris also said, that he understood the impression had got abroad that this society was simply one for the western and middle part of the State. Now in regard to this, he wanted it distinctly understood, that such was not the case. That it was not an Eastern or Western society. But just what its name implied. *A New York State Society.* Its cause, being to try and benefit every qualified Veterinary Surgeon, in the State of New York, whether a resident of one of the large cities of New York or Brooklyn, or a remote village of the interior. Its intentions, to get all interested and all work together for one grand cause, to elevate the profession to its proper standing, so it will become better recognized by the Local and National Governments, and by the public at large.

The Secretary then read a synopsis of the last meeting, which were approved by the society.

In the absence of Prof. Jas. Law, who came in later, President Morris made a report on what had been done pertaining to Legislation. He said there had been seventy-six votes cast in favor of the bill, when eighty-four votes would have passed the bill, showing that the labors of the committee had been effectual, to a certain extent, and that we ought to keep pushing the matter.

He also repeated that the object in going to New York was to get the assistance of the veterinary profession, of the eastern part of the State, in getting this bill through the Legislature. That there was no reason why it should not be supported from a sense of merit, as it would oblige men who wish to enter the profession after its passage, to do so through a college or university, granting veterinary degrees.

President Morris then made a motion that a resolution of thanks be offered, by the members of the society, to the Hon. Rufus S. Peck, for the services rendered in introducing the bill, and supporting same when brought up for action. Prof. Jas. Law, seconded the motion, voted on, and carried unanimously.

Following is the resolution:

*Resolved,* That it is the sense of the New York State Veterinary Medical Society, that individually, they owe a debt of gratitude and appreciate the valuable services rendered by the Hon. Rufus S. Peck, of Cortlandt, for his untiring energy and fidelity in behalf of the Veterinary Surgeons' bill, which he so ably farthered during the last session of the State Legislature, and that we, the representative Veterinary Surgeons' of the State of New York, feel under personal obligations to Mr. Peck, and that we deem it a privilege to express the same to him.

President Morris then said that if any members of the profession, who were present, wished to discuss the merits of the bill, they were at liberty to do so. Quite a discussion followed regarding changes in certain clauses, which were amicably settled.

Prof. A. Liautard suggested that a meeting of all the qualified Veterinarians in New York State be called, for the purpose of making every one acquainted with the merits of the bill. He also suggested that seven members of the Board of Examiners, be chosen from the members of the New York State Veterinary Medical Society, and eight from the members of the profession at large, who hold diplomas from a college or university granting veterinary degrees.

This was made a motion by Prof. Law, and seconded by Dr. Jno. Wende, voted on and carried. The discussion was then continued by Prof. Liautard, Prof. Law, Roscoe R. Bell, D. V. S., of Brooklyn, and several other prominent members of the profession.

At the close of the discussion all agreed that the bill was all that could be desired at present, and that it would meet the approval of the profession at large, and that each and every one of the qualified veterinary surgeons throughout the State, use his personal influence and energy to secure the passage of same.

Adjournment was then taken for dinner.

President Morris called the meeting to order at 2:30 P. M.

The following applications for membership were received by the Censors and placed on file :

R. R. Bell, D. V. S., Brooklyn, N. Y.; Geo H. Berns, D.V.S., Brooklyn, N.Y.; R. S. Huidekoper, M.D.V.S. New York; W. H. Conklin, D.V.S. New York; H. McWhinnie, D.V.S., Troy, N. Y.; Wm. Machon, V.S., New York; L. McLeon, M.R.C.V.S., Brooklyn, N. Y.; Wm. Somerville, D.V.S., Buffalo, N. Y.; G. B. Ackerman, D.V.S., New York; C. B. Comstock, D.V.S., Brooklyn, N. Y.; L. Willyoung, Albion, N. Y.

Prof. A. Liautard was made an Honorary Member by a unanimous vote.

A resolution of thanks was tendered the visiting members of the profession, for the interest they had taken in the matters brought before the meeting, and all were asked to become members of the Society.

President Morris then read a paper on "Tetanus."*

A lively discussion on Tetanus followed, which was entered into by all members of the profession present.

Prof. Law, said that at present there was no doubt as to the germ theory, and stated his experience in several cases. He also said that some cases were known to have recovered without any treatment, while other cases succumbed to this fatal disease with the best medical treatment.

---

* See page 439, this number of THE JOURNAL.

Prof. Liautard was also of the opinion that the disease was caused by the Bacillus Tetani, and when possible, large doses of purgatives, with antiseptic treatment to the wound in traumatic cases, and perfect quietude are demanded.

Prof. Law thought the early use of slings was a great benefit.

Dr. Geo. H. Berns stated his experience in citing seventeen cases he had at one time, which he treated with large doses of purgatives, and one-ounce doses of tartar emetic in the drinking water three times daily, for about a week, then alternated with one-ounce doses of powd. lobelia, and antiseptic treatment of wounds, quietude and slings. And he stated he was successful in making a good recovery of thirteen cases out of the seventeen.

Dr. McWhinnie cited a case which made a good recovery by blistering and hot poultices, applied to the spine and mastoid muscles.

Dr. Roscoe R. Bell said, that although he did not have the affidavit for this, he thought it could be shown to be true, that a horse suffering from a severe attack of tetanus, while being taken from the owner's stable to a veterinary hospital in an ambulance, while passing under the Elevated Railroad, became frightened, and either jumped or fell out of the ambulance to the pavement, and immediately got up and walked home, evidently cured by the shock.

Dr. Willyoung said he had good success by slinging his patient about three or four days and giving large doses of purgatives, followed by large, and oft-repeated doses of solid or fluid extract of cannibis indica; so far he had not lost a case out of seven or eight he had treated.

Dr. Wm. Somerville, Jr., thought that the best treatment was perfect quietude, allowing no one to disturb the patient and giving no medicine except a brisk purgative to clean out the alimentary track, and allow nature to do the rest, he had treated cases both with this form of treatment, and with the many ways laid down in our text books, but had better success by leaving the animal to wonderful *vis medicatrix naturæ.*

Prof. Law said that animals left alone would on the fourth or fifth day be found to be perspiring very much and do so for from twelve to twenty-four hours, when the spasms of the muscles would appear to be considerably relaxed and better, and thought that hot water baths would be beneficial.

Dr. N. P. Hinkley related the history of cases he had treated with steam baths, and stated that, same as in other modes of treatment, some of the patients recovered and others died.

Several other gentlemen continued the discussion of Dr. Morris's paper which brought out several bright ideas regarding the cause and treatment of tetanus, and it was quite late in the afternoon when the discussion closed. As this was only a one day session, and most of the gentlemen present were obliged to return home by the early trains, the reading of the other papers was postponed until the next meeting.

The meeting was then adjourned until the second week in January, of 1892, subject to a call from the Secretary.

NELSON P. HINKLEY, *Secretary.*

